# A rapid, simple, and highly efficient method for VIGS and in vitro-inoculation of plant virus by INABS applied to crops that develop axillary buds and can survive from cuttings

**DOI:** 10.1186/s12870-021-03331-9

**Published:** 2021-11-20

**Authors:** Qili Liu, Kedong Xu, Lun Yi, Yalin Hou, Dongxiao Li, Haiyan Hu, Feng Zhou, Puwen Song, Yongang Yu, Qichao Wei, Yuanyuan Guan, Ping Hu, Ruifang Bu, Eryong Chen, Xiaojia Su, Honglian Li, Chengwei Li

**Affiliations:** 1 College of Plant Protection, Henan Agricultural University, Zhengzhou, 450002 China; 2 Postdoctoral Research Base, Henan Institute of Science and Technology, Xinxiang, 453001 China; 3 Henan Engineering Research Center of Crop Genome Editing, Henan Institute of Science and Technology, Xinxiang, 453001 China; 4 Key Laboratory of Plant Genetics and Molecular Breeding, Zhoukou Normal University, Zhoukou, 466000 China; 5 College of Biological Engineering, Henan University of Technology, Zhengzhou, 450001 China

**Keywords:** Virus-induced gene silencing (VIGS), In vitro-inoculation of plant virus, Agrobacterium-based infiltration, Injection of no-apical-bud stem sections (INABS)

## Abstract

**Background:**

Virus-induced gene silencing (VIGS) is one of the most convenient and powerful methods of reverse genetics. In vitro-inoculation of plant virus is an important method for studying the interactions between viruses and plants. *Agrobacterium*-based infiltration has been widely adopted as a tool for VIGS and in vitro-inoculation of plant virus. Most agrobacterium-based infiltration methods applied to VIGS and virus inoculation have the characteristics of low transformation efficiencies, long plant growth time, large amounts of plant tissue, large test spaces, and complex preparation procedures. Therefore, a rapid, simple, economical, and highly efficient VIGS and virus inoculation method is in need. Previous studies have shown that the selection of suitable plant tissues and inoculation sites is the key to successful infection.

**Results:**

In this study, *Tobacco rattle virus* (TRV) mediated VIGS and *Tomato yellow leaf curl virus* (TYLCV) for virus inoculation were developed in tomato plants based on the *agrobacterium tumefaciens*-based infiltration by injection of the no-apical-bud stem section (INABS). The no-apical-bud stem section had a “Y- type” asymmetric structure and contained an axillary bud that was about 1–3 cm in length. This protocol provides high transformation (56.7%) and inoculation efficiency (68.3%), which generates VIGS transformants or diseased plants in a very short period (8 dpi). Moreover, it greatly reduces the required experimental space. This method will facilitate functional genomic studies and large-scale disease resistance screening.

**Conclusions:**

Overall, a rapid, simple, and highly efficient method for VIGS and virus inoculation by INABS was developed in tomato. It was reasonable to believe that it can be used as a reference for the other virus inoculation methods and for the application of VIGS to other crops (such as sweet potato, potato, cassava and tobacco) that develop axillary buds and can survive from cuttings.

**Supplementary Information:**

The online version contains supplementary material available at 10.1186/s12870-021-03331-9.

## Background

Virus-induced gene silencing (VIGS) is one of the most convenient and powerful methods of reverse genetics [1], and it is increasingly widely used to study plant gene functions [2, 3]. Several viruses have been developed for use in VIGS [2, 4]. One of these viruses, the *Tobacco rattle virus* (TRV), has a broad host range and has been used in VIGS as a vector to study gene function in many species, including *Solanum* spp. [5–9], *Nicotiana* spp. [10–12], *Arabidopsis thaliana* [13], *Papaver somniferum* [14], *Gossypium hirsutum* [15], *Amaranthus tricolor* [16], *Triticum aestivum*, and *Zea mays* L. [17]. The molecular mechanisms of VIGS have been well studied, and the development and improvement of VIGS is presently focused on the creation of new viral constructs for different plant species, the search for new reporter genes to control VIGS efficiency, and the development of new, efficient infection methods [1].

In vitro-inoculation of plant virus is an important method for studying the interaction between viruses and plants. *Agrobacterium*-based infiltration has been widely used as an inoculation tool for the infectious clones of plant viruses. The *Tomato yellow leaf curl virus* (TYLCV) belongs to the genus *Begomovirus*, within the family *Geminiviridae.* It is distributed worldwide and rated as the third most important plant virus [18]. TYLCV is a typical member of geminiviruses, and its inoculation methods are similar to those of most DNA viruses. Several methods have been developed to test the infectivity of TYLCV and to understand the mechanisms of TYLCV resistance in plants; these include natural field infection, whitefly inoculation in cages, leaf or stem agroinfiltration, and biolistic inoculation [19–21].

However, most methods used for VIGS infection and inoculation of DNA virus have low transformation efficiency and require long plant growth times, large amounts of plant tissue, large test spaces, and complex preparation procedures. *Agrobacterium tumefaciens*-based infiltration is often used for VIGS and mechanical inoculation for virus inoculation, but it typically takes a long time for symptoms to appear [22]. Studies have shown that the selection of suitable plant tissues and inoculation sites is the key to successful infection. Commonly used tissues include the meristems of seedlings [23], three-week-old micro-shoots [24], and the stems or petioles of 4–6 leaf stage plants [25]. However, the use of such sites is complicated and time-consuming, increasing the operational complexity of inoculation [23–25]. Therefore, a rapid, simple, economical, and highly efficient VIGS and virus inoculation method is in need. In this study, TRV mediated VIGS and TYLCV for virus inoculation were developed in tomato plants based on the *agrobacterium tumefaciens*-based infiltration by injection of the no-apical-bud stem section (INABS). This protocol gives a high transformation and inoculation efficiency and can generate transformants or diseased plants in a very short period of time. Moreover, it greatly reduces the required experimental space. This method will facilitate functional genomics studies and large-scale disease resistance screens.

## Results

### The no-apical-bud stem section is optimal for VIGS and in vitro-inoculation of plant virus

#### VIGS

**Fig. 1:**
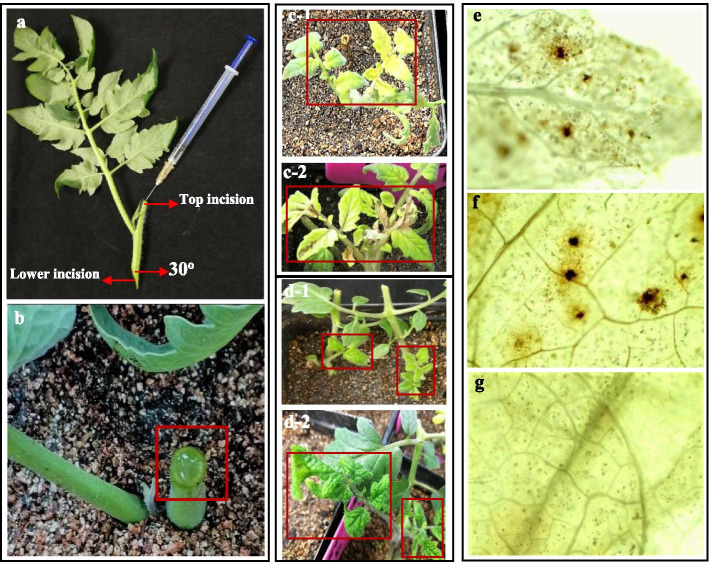
Injection of no-apical-bud stem sections (INABS) for tomato. **a** the tomato no-apical-bud stem section and *Agrobacterium* injection; **b** a film of agroinfiltration liquid formed at the top incision of the injected bare stem; **c-1** and **c-2** the grown tomato axillary buds transformed with TRV-*SlPDS* (*A. tumefaciens* carrying pTRV2-*SlPDS* and pTRV1) showed leaf bleaching; **d-1** and **d-2** the axillary buds of tomato showed obvious TYLCV infection symptoms; **e–g** H_2_O_2_ production was detected in leaves that emerged from the axillary bud of the no-apical-bud stem sections infected with TRV-*SlPDS* (**e**) and TYLCV (**f**); **g** WT control groups for (**e**) and (**f**)

**Fig. 2:**
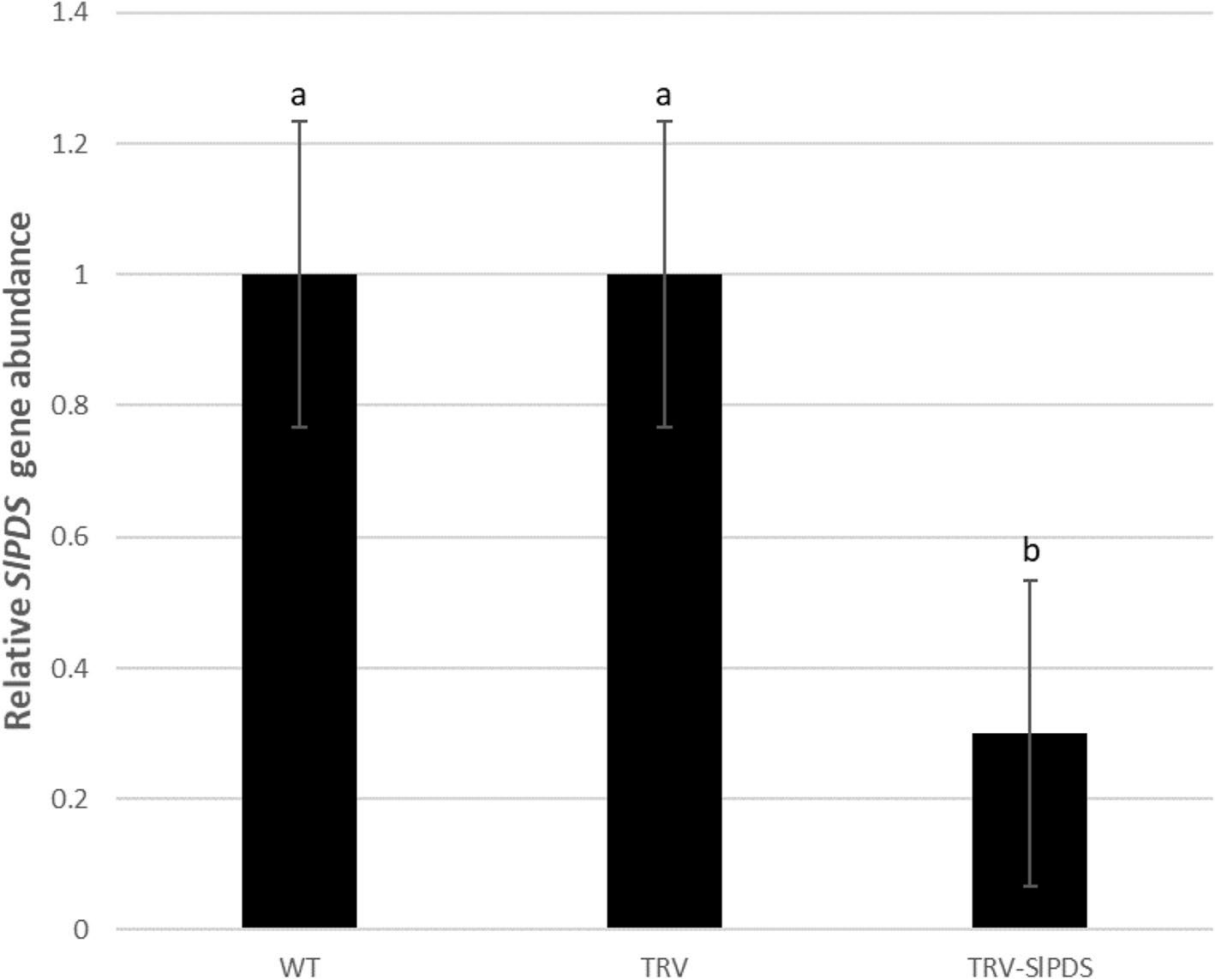
Quantitative qRT-PCR analysis of TRV-mediated VIGS of the *PDS* gene in tomato leaves by injection of the no-apical-bud stem section (INABS). WT: wild-type plants; TRV: VIGS control plants infected by *Agrobacterium tumefaciens* carrying pTRV 2 and pTRV1; TRV-*SlPDS*: *PDS*-silenced plants infected by *A. tumefaciens* carrying pTRV2-*SlPDS* and pTRV1. Values (means ± SD are the averages of three independent experiments, and significant differences (*P* ≤ 0.05) among treatments in each group are indicated by different letters (Duncan’s multiple range test)

The phytoene desaturase (*PDS*) gene as a reporter gene proved the efficacy of the TRV mediated VIGS system by INABS in tomato. About 100–200 μl of agroinfiltration liquid harboring empty vector control TRV (*A. tumefaciens* carrying pTRV2 and pTRV1), treatment TRV-*SlPDS* (*A. tumefaciens* carrying pTRV2-*SlPDS* and pTRV1) could be slowly injected into the bare stem of no-apical-bud stem section using a plastic syringe and needle (Fig. 1a). A film of agroinfiltration liquid formed at the top of the injected stem sections when infiltration liquid had filled the entire bare stem (Fig. 1b). No-apical-bud stem sections without agrobacterium injection were used as wild-type (WT) controls. Six days after inoculation, bleaching started to become evident in portions of the mesophyll tissue in the axillary buds transformed with TRV-*SlPDS*. About 10 days after inoculation, the axillary buds of treatment TRV-*SlPDS* had grown out, and widespread bleaching was evident in the grown leaves (Fig. 1c-1 and c-2). Bleaching of mesophyll tissue were not observed in TRV and WT control. H_2_O_2_ production increased in leaves that emerged from the axillary bud of the no-apical-bud stem section of treatment TRV-*SlPDS* (Fig. 1e). The expression of* PDS* gene of the grown bleaching leaves treated with TRV-*SlPDS* was significantly down-regulated at 8 days after inoculation (Fig. 2). The grown axillary buds of treatment TRV*-SlPDS* showed a high gene silencing success rate (56.7%, Table 1) based on qRT–PCR analysis.

**Table 1:** Effects of bacterial concentration and inoculation time on the efficiency of INABS infiltration

Inoculation time	VIGS efficiency of the *PDS* gene (%) and TYLCV inoculation success rate (%) after infiltration of *Agrobacterium* at different concentrations		
	0.5 (OD_600_)	1.0 (OD_600_)	1.5 (OD_600_)
4 dpi 8 dpi 12 dpi	11.3b/16.7b	17.7c/27.7b	16.0b/23.3b
	21.7a/34.3a	56.7a/68.3a	30.7a/56.0a
	17.7b/29.3a	45.0b/61.0a	28.3a/55.0a

*Note*: Each value represents the mean transformation efficiency or success rate of virus infection (*n* = 3). Within a column, significant differences (*P* ≤ 0.05) among treatments are indicated by different letters (Duncan’s multiple range test)

#### Virus inoculation in vitro

The INABS method achieved the infection of DNA virus. In the case of an important DNA virus, TYLCV was inoculated into tomato no-apical-bud stem sections as described above, and TYLCV infection symptoms appeared 3 days after inoculation. The young leaves of axillary buds began to show crinkling and yellowing. At 10 ~ 12 dpi, the axillary buds had grown out and showed obvious TYLCV infection symptoms (Fig. 1 d-1 and d-2). Consistent with this observation, H_2_O_2_ production increased in leaves that had emerged from the axillary buds of INABS—a known response of plant cells to virus infection (Fig. 1 f). qRT–PCR analysis showed that the young leaves were infected by TYLCV and the incidence of TYLCV diseases was 27.7, 68.3, 61.0% at 4 dpi, 8 dpi and 12 dpi respectively (Table 1). H_2_O_2_ and TYLCV were not detected in the young leaves of WT control, indicating the no-apical-bud stem sections were not inoculated with TYLCV.

### Optimization of bacterial optical density (OD) and INABS time

Bacterial OD and INABS time were optimized. Using different combinations of INABS time points (4, 8, and 12 d) and bacterial concentrations (OD600 of 0.5, 1.0, and 1.5), an agroinfiltration liquid with OD600 of 1.0 and an INABS time point of 8 d resulted in significantly higher VIGS efficiency (56.7%) and TYLCV inoculation rate (68.3%) by symptoms observation and qRT–PCR analysis (Table 1). An OD600 of 1.0 and an INABS time of 8 d were therefore adopted to achieve the highest transformation efficiency and success rate of virus inoculation (Table 1).

### INABS promotes faster virus infection and VIGS process

Parallel *Agrobacterium*-mediated virus infection and VIGS experiments using three different inoculation methods (INABS, infiltration of the dorsal leaf and injection of the basal stem) showed that the INABS method outperformed the other inoculation methods and sites (Table 2). Compared with the dorsal leaf or stem base, the no-apical-bud stem section could hold a higher volume of agroinfiltration liquid (100–200 μl), thus increasing the infection success rate. The INABS method developed here required only 10–12 d and 8–10 d for the appearance of leaf bleaching and disease symptoms, respectively. It produced symptoms much more rapidly than the other two methods: infiltration of the dorsal leaf (30–50 d and 23–45 d) and injection of the basal stem (35–60 d and 30–45 d).

**Table 2:** Comparison of the virus infection and VIGS process of three inoculation methods

Inoculation methods	Inoculation sites	Volume of agroinfiltration liquid that can enter plants (μl)	Time required to obtain VIGS plants (d)	Success rate of VIGS (%)	Time required to obtain symptomatic plants (d)	Inoculation success rate of TYLCV (%)
**INABS**	**no-apical-bud stem section**	**100–200**	**10–12**	**60**	**8–10**	**72**
Infiltration the dorsal leaf	back of tender leaf	10–50	30–50	30	23–45	40
**Injection of the basal stem**	**base of stem (~ 2–3 cm from the soil surface)**	**20–30**	**35–60**	**36**	**30–45**	**48**

### INABS rapidly identifies TYLCV-resistant tomato varieties

**Fig. 3:**
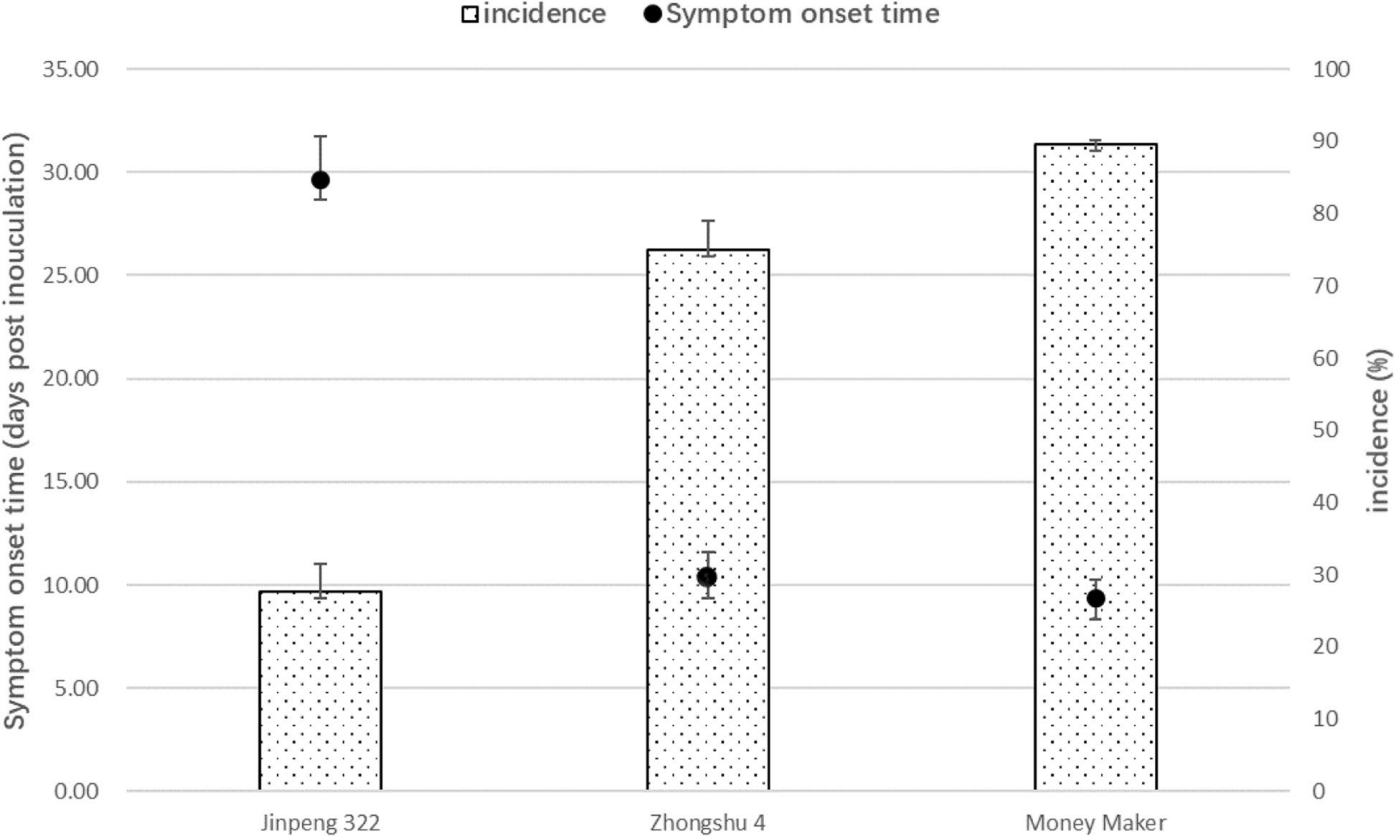
A rapid TYLCV resistance assay (incidence and symptom onset time) based on the injection of no-apical-bud stem sections (INABS) from three tomato varieties, ‘Jinpeng 322,’ ‘Zhongshu 4,’ and ‘Money Maker.’ Values (mean ± SD are the averages of three independent experiments, and significant differences (*P* ≤ 0.05) among varieties are indicated by different letters

We next tested whether the INABS method could be used for rapid assessment of TYLCV resistance using three tomato varieties with different degrees of resistance to TYLCV: ‘Money Maker’ (with TYLCV-susceptible tomato varieties), ‘Zhongshu 4’ (with general susceptibility to TYLCV), and ‘Jinpeng 322’ (with highly TYLCV-resistant variety). The three varieties showed different levels of resistance to TYLCV infection in our experiment (Fig. 3). ‘Jinpeng 322’ took the longest time to show symptoms (~ 27 dpi) and showed high resistance to TYLCV (27.7% incidence). ‘Zhongshu 4’ showed symptoms after 10 days and had an incidence of 75.0%. ‘Money Maker’ showed high susceptibility (89.7% incidence, symptoms ~ 9 days after inoculation). In addition to identify the varietal susceptibility to TYLCV, INABS also greatly reduced the required experimental space and permitted disease resistance evaluation at the seedling stage.

## Discussion

In this study, a rapid, simple, and highly efficient method for INABS was developed for VIGS and plant DNA virus inoculation in tomato. The method greatly facilitates VIGS progress and offers a feasible strategy for identifying TYLCV-resistant varieties that are suitable for inoculation in VIGS and for the initial screening of DNA virus-resistant plants under controlled conditions. We also observed that agroinfiltrated stem sections with water films had a higher efficiency than those without water films (data not shown). This may be because the water film persisting for a certain period of time was filled with agroinfiltration liquid and allowed for more efficient infiltration of the epidermal cells, thereby increasing the ratio of infiltrated cells. Compared to other traditional methods of inoculation, this rapid assay has many advantages, including the rapid onset of viral symptoms, relatively short plant growth time, reduced space requirements, and the ability to screen for TYLCV resistance in tomato. It is also could be a useful tool for studying viral biology in tomato and other crops (such as sweet potato, potato, cassava and tobacco) that develop axillary buds and can survive from cuttings.

There are many ways to improve the efficiency of VIGS. Examples include infiltration of lower leaves with a needleless syringe [26], pouring inoculum from the apical region and soaking wounded stems and leaves randomly pierced with a sterilized needle [16], infiltration of the abaxial side of both cotyledons [9, 15], vacuum and co-cultivation agroinfiltration of germinated seeds [17], carpopodia of young fruit attached to the plant after pollination [5], soil adjacent to the plant roots (this method is called “agrodrenching”) [10] and infiltration at three different stages of seedling development [14]. It typically requires ~ 15–60 days to obtain transformants with these methods [1, 17]. With the INABS method, the time period from inoculation to obtaining the target plants was reduced greatly (~ 10–12 days). The injected stems with water films formed at the top incision of the injected bare stem exhibited a higher transformation efficiency than those without water films. It may be that the agroinfiltration water film provides extra volume that allows more time for the *Agrobacteria* to enter and infect the axillary buds. Compared with other previously reported methods, INABS has a relatively higher VIGS efficiency and infection efficiency of TYLCV. In addition, compared with other infiltration sites, the no-apical-bud stem was more suitable for inoculation and virus infection because it could accept a high volume of agroinfiltration liquid, which rapidly entered the susceptible young axillary buds.

The mechanical inoculation of viruses into plants has been studied for years. Plant viruses are typically inoculated by rubbing an inoculant onto the surface of the leaf: plant material known or suspected to be virus infected, a solution of virus preparation, or an infectious clone [21]. Inoculation with a DNA virus usually requires a needle to inject bacterial cultures harboring an infectious clone of the virus into the stems or petioles of plants at the 4–6 leaf stage [25]. Here, injection of no-apical-bud stem sections induced infection symptoms in a short period of time, probably because axillary buds contain much younger cells that are more susceptible to virus infection. Therefore, no-apical-bud stems are well-suited for virus inoculation compared with other plant tissues that are mechanically inoculated using previously described methods.

The artificial pruned axillary buds, which affects the growth rate, and the susceptibility of young leaves to the virus are two key success factors for INABS. The growth rate of axillary buds is regulated by plant hormones. Sae et al. proposed a regulatory mechanism by which plant hormones control apical dominance [27]. When apical buds are present, indoleacetic acid (IAA) derived from apical buds activates IAA-inducible genes and represses the activity of isopentenyl transferase (IPT) in nodes, indirectly promoting abscisic acid (ABA) biosynthesis in nodes and axillary buds. ABA then activates the expression of ABA-inducible genes, inhibiting the outgrowth of axillary buds. Decapitation removes the IAA supply from apical buds, causing IAA deficiency in the nodes. *IPT* is expressed in nodes and is involved in cytokinin production. Cytokinin from the nodes then enters the axillary buds and promotes their outgrowth. The no-apical-bud stem section has a “Y-type” asymmetric structure that enriches cytokinin in the axillary buds, and the lateral leaf performs photosynthesis to provide energy for the axillary buds. The “Y” structure ensures the survival of no-apical-bud stems and facilitates the virus infection of axillary buds. Based on the general principles of INABS, it is reasonable to believe that it can be applied to other crops that develop axillary buds and can survive from cuttings (such as sweet potato, potato, cassava and tobacco).

## Conclusion

Overall, INABS was developed as a rapid, simple, and highly efficient method for VIGS and virus inoculation in tomato. The core of this method was the no-apical-bud stem section with a “Y- type” asymmetric structure and an axillary bud that was about 1–3 cm in length. This protocol provides high transformation and inoculation efficiency, which generates VIGS transformants (only 10–12 d for leaf bleaching) or diseased plants in a very short period (8–10 d) respectively. Moreover, it greatly reduces the required experimental space. We inferred that the method can also be used for other virus inoculation in vitro and VIGS applied to other crops (such as sweet potato, potato, cassava and tobacco) that develop axillary buds and can survive from cuttings. This method will facilitate functional genomic studies and large-scale disease resistance screening.

## Methods

### Plant materials and selection of the no-apical-bud stem section

The cultivated *Solanum lycopersicum* (tomato) variety ‘Money Maker,’ which is commonly used in genetic research, was planted in soil and grown in a greenhouse at 25–28 °C for 1 month until the stems were about 35 cm in length.

**Fig. 4:**
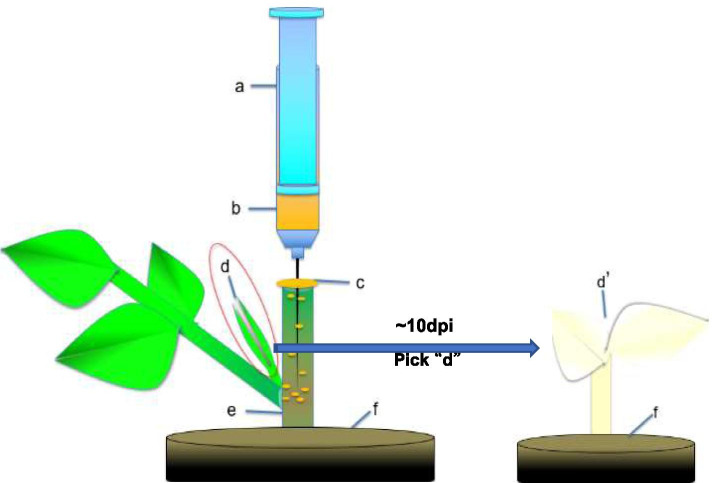
The infiltration of the no-apical-bud stem section (INABS) method. **a** A syringe. **b** The agroinfiltration liquid. **c** An agroinfiltration water film formed at the top incision of the injected bare stem. **d** An axillary bud. **e** A no-apical-bud stem section. **f** Nutritional soil. d’: An axillary bud showing infection symptoms

Stem sections (~ 4–6 cm) with no apical buds were removed and planted in nutritional soil (2:1 mixture of moss peat and vermiculite, PindStrup, Denmark) (Fig. 4 d and e) for 1–2 d before agroinfiltration. The no-apical-bud stem section had a “Y- type” asymmetric structure, and their upper incision was located about 2.5–3.5 cm from the base of the lateral branch or compound leaf. The top incision of each section was flat, whereas the lower incision was made at a 30° angle. Each stem section contained an axillary bud that was about 1–3 cm in length (Fig. 4 d).

### VIGS and in vitro-inoculation of plant virus by INABS

#### VIGS

The phytoene desaturase (*PDS*) gene was used to verify the efficacy of the TRV-VIGS system because of its ability to produce a distinct bleaching phenotype. Treatment TRV-*SlPDS*, TRV (empty vector control), and WT (no-apical-bud stem sections without agrobacterium injection) were set up, and each treatment had one-hundred no-apical-bud stem sections. The pTRV1, pTRV2, and pTRV2-*SlPDS* constructs were transformed into *A. tumefaciens* strain GV3101 for further transient transformation in tomato (for vector construction details, refer to [8]). Bacterial clones carrying the constructs were cultivated overnight at 28 °C, harvested when OD600 reached 1.5–2.0, centrifuged at 4000 rpm for 10 min, and re-suspended in an agroinfiltration liquid to the appropriate concentration for agroinfiltration. A 1:1 (v/v) mixture of pTRV1 and pTRV2-*SlPDS* agroinfiltration suspensions was prepared as TRV-*SlPDS* treatment.

Different *Agrobacterium* concentrations (OD600 0.5, 1.0, and 1.5) were tested to determine the optimal concentration for efficient transformation. In each batch, no-apical-bud stem sections inoculated with an empty vector TRV (a 1:1 mixture of pTRV1 and pTRV2 agroinfiltration liquids) served as empty vector controls. The infiltration solution was prepared as follows: 41.65 mM D-glucose, 100 mM CaCl_2_, 100 mM MES-KOH (pH 5.6) stock solution, 0.011μM BAP, 0.01% Silwet L-77, 0.05 mM MgCl_2_, and 12.5 mM AS stock solution (made with dimethylformamide, DMF). Double-distilled H_2_O was used to adjust the final volume to 20 ml [28].

#### Virus inoculation in vitro

The infectious vector TYLCV-[CN:SH2] (Refer to [29] for details on vector construction) and the binary vector pBINPLUS (negative control) were transferred into *A. tumefaciens* strain EHA105 by electroporation. EHA105 clones carrying the aforementioned constructs were cultivated for 24 h at 28 °C in Luria-Bertani (LB) medium supplemented with appropriate antibiotics, harvested by centrifugation, and resuspended to final OD600 values of 0.5, 1.0, and 1.5 in liquid Murashige and Skoog (MS) medium supplemented with 100 μM acetosyringone [24]. Different bacterial concentrations were tested to determine the optimal concentration for high transformation efficiency. Different concentrations of agroinfiltration liquid harboring the infectious clone were then injected into tomato no-apical-bud stem sections using 5-ml syringes (Fig. 4 a-e). No-apical-bud stem sections without agrobacterium injection were used as WT controls. Two treatments had one-hundred no-apical-bud stem sections respectively.

#### Experimental operation details

Agroinfiltration liquid for VIGS (TRV-*SlPDS*, TRV) or for virus inoculation in vitro (TYLCV-[CN:SH2]) was injected into tomato no-apical-bud stem sections. The needle was inserted into the upper end of the top incision, 0.5–1 ml of the bacterial liquid was injected, and the needle was held in place for 1–2 min. The needle was pulled out slowly, and the bacterial liquid naturally formed a liquid film at the incision site (Fig. 4 c). The growth and symptoms of no-apical-bud stem sections were observed daily. The growing axillary buds were measured for VIGS efficiency and TYLCV inoculation success rate every 4 days based on symptoms and qRT-PCR anylysis (4, 8, and 12 d) after injection to evaluate the effects of transformation and inoculation.

### Comparison of different inoculation methods and sites

Agroinfiltration liquid containing TRV (OD600 1.0, empty vector control of VIGS), TRV-*SlPDS* (OD600 1.0, treatment of VIGS) or TYLCV (OD600 1.0, treatment of virus inoculation in vitro) was injected into different parts of the tomato plants respectively: the no-apical-bud stem section, the dorsal leaf, or the basal stem. The no-apical-bud stem sections without any agrobacterium injection or infiltration were used as WT controls. Each treatment was replicated one-hundred times. Three biological repetitions were developed.

#### INABS

The injection of the no-apical-bud stem section is described above.

#### Infiltration of the dorsal leaf

Tomato seedlings at the three-true-leaf stage were used for dorsal leaf *Agrobacterium* infiltration. The agroinfiltration liquid was infiltrated into the back of the tomato leaves using 5-ml syringes without needles.

#### Injection of the basal stem

Tomato seedlings at the three-true-leaf stage were used for basal stem *Agrobacterium* infiltration using 5-ml syringes with needles. Three sites on each stem were infiltrated.

After infiltration, the plants were grown in darkness for 48 h at 25–28 °C with a relative humidity of 80–90%. Thereafter, the stem segments were cultured in a pest-controlled greenhouse at 25–28 °C with 70% relative humidity and a 16 h/8 h light/dark photoperiod. The symptoms of VIGS and infection of TYLCV were recorded and evaluated.

### Total RNA and DNA extraction and qRT–PCR analysis

Total RNA was extracted from the leaves of the wild type (non-transgenic control), pTRV1 and pTRV2 (empty vector controls), and symptomatic plants using the TRIzol reagent (Invitrogen, USA) according to the manufacturer’s instructions. First-strand cDNA was synthesized using mixtures containing 2 mg of total RNA, oligo (dT), and M-MLV Reverse Transcriptase (Promega, USA) according to the manufacturer’s instructions. The cDNAs were then used as templates for quantitative RT–PCR (qRT–PCR) with gene-specific primers outside the gene regions targeted for silencing.

Total DNA was extracted from leaves that grew from axillary buds inoculated by TYLCV. qRT–PCR was performed with SYBR Premix Ex Taq (TaKaRa, China) using an ABI Prism 7500 instrument (Applied Biosystems, USA) following the manufacturers’ recommendations. The primers used for qRT–PCR are listed in supplementary materials (Table S1).

### H_2_O_2_ determination

Inoculated leaves were stained with 3,3-diaminobenzidine (DAB) [30] to examine the distribution and level of H_2_O_2_. Tomato leaves were excised from plants and placed into 20-ml tubes, covered with a DAB-HCl solution (1 mg/ml, pH 3.8), and incubated in a growth chamber for 8 h at 25 °C. When the red-brown DAB solution moved to the top of leaves via the veins, the chlorophyll of whole blades was removed by immersing the samples directly in a fixative solution (anhydrous ethanol: acetic acid = 3:1) for 24 h. Leaves staining was developed with hydrated trichloroacetaldehyde three times and observed under an optical microscope (Olympus, Japan).

### Identification of TYLCV-resistant tomato varieties

Seeds of the TYLCV-susceptible tomato varieties ‘Money Maker’, ‘Zhongshu 4’ (with general susceptibility to TYLCV; provided by the Zhengzhou Hongfeng Seed Co.) and the highly TYLCV-resistant variety ‘Jinpeng 322’ (provided by the Xi’an Jinpeng Seed Co.) were germinated in a growth chamber (25 °C, cool white fluorescent lights, 50–100 μEm^− 2^ s^− 1^, 16 h light/8 h dark photoperiod). After 1 month, when the stems were ~ 35 cm in length, two-hundred no-apical-bud stem sections were harvested for each variety (one half was used for inoculation of TYLCV and the other half was used as WT control), and virus inoculation experiments were performed.

## Supplementary Information

**Additional file 1: Table S1.** Primers used for quantitative real-time fluorescence PCR (qRT-PCR).

## Data Availability

All data generated or analyzed during this study are included in this published article and its supplementary information files. The datasets used and/or analyzed during the current study are available from the corresponding author on reasonable request.

## Abbreviations

VIGS: Virus-induced gene silencing
INABS: Injection of no-apical-bud stem sections
TRV: *Tobacco rattle virus*
TYLCV: *Tomato yellow leaf curl virus*
*PDS*: Phytoene desaturase
OD: Optical density
IAA: Indoleacetic acid
IPT: Isopentenyl transferase
ABA: Abscisic acid
LB: Luria-Bertani
MS: Murashige and Skoog
DAB: 3,3-diaminobenzidine
