# Supplementary material for: A rapid, simple, and highly efficient method for VIGS and in vitro-inoculation of plant virus by INABS applied to crops that develop axillary buds and can survive from cuttings
Source: BMC Plant Biol. 2021 Nov 20;21:545. doi: 10.1186/s12870-021-03331-9 (PMC8605592; doi:10.1186/s12870-021-03331-9)
Supplement: Supplementary file 1 — Additional file 1: Table S1. Primers used for quantitative real-time fluorescence PCR (qRT-PCR). [file 12870_2021_3331_MOESM1_ESM.docx]

| Primer name | Primer sequence (5’-3’) | Size of PCR products (bp) | Application |
| --- | --- | --- | --- |
| **sIPDS-F** | **CGGGGTACCGGCACTCAACTT TATAAACC** | **416** | **Detect *Solanum lycopersicum* phytoene desaturase (pds), mRNA** |
| **sIPDS-R** | **CGGGGATCCTTCAGTTTTCTG TCAAACC** |  |  |
| prTRV2 CP-F | CTGAATCACTTGCGCTAAT | 660 | Detect if the pTRV2 was transformed into the plants |
| prTRV2 CP-R | CAGAGTTCACGTCCTTAAA |  |  |
| **prTRV192 RdRp-F** | **TCTTCACAGCCTGTATGACT** | **256** | **Detect if the pTRV1 was transformed into the plants** |
| **prTRV192 RdRp-R** | **TAGAACACATTCCCTTGGTA** |  |  |
| TYLCV-YG-3 | GAGTTCCCCTGTGCGTGAA | 139 | Detect TYLCV by qRT-PCR |
| TYLCV-YG-4 | CTGTTCGCAAGTATCAATCAA GGT |  |  |
| **Tomato25s-Rrna-UNIV (+)** | **ATAACCGCATCAGGTCTCCA** | **113** | **Detect tomato 25s rRNA by qRT-PCR** |
| **Tomato25s-Rrna-UNIV (-)** | **CCGAAGTTACGGATCCATTT** |  |  |

**Table S1.** Primers used for quantitative real-time fluorescence PCR (qRT-PCR)
